# Effects of Exposure to Dietary Chromium on Tissue Mineral Contents in Rats Fed Diets with Fiber

**DOI:** 10.1007/s12011-014-9973-z

**Published:** 2014-04-22

**Authors:** Anna Prescha, Monika Krzysik, Katarzyna Zabłocka-Słowińska, Halina Grajeta

**Affiliations:** Department of Food Science and Dietetics, Wroclaw Medical University, Borowska 211, 50-556 Wroclaw, Poland

**Keywords:** Cellulose, Pectin, Chromium, Calcium, Magnesium, Iron, Zinc, Rats

## Abstract

This study evaluated the effects of diets with fiber (cellulose and/or pectin) supplemented with chromium(III) on homeostasis of selected minerals in femurs, thigh muscles, livers, and kidneys of rats. For 6 weeks, male rats were fed experimental diets: a fiber-free diet (FF), a diet containing 5 % cellulose (CEL), 5 % pectin (PEC), or 2.5 % cellulose and 2.5 % pectin (CEL + PEC). These diets had 2.53 or 0.164 mg Cr/kg diet. The tissue levels of Ca, Mg, Zn, Fe, and Cr were determined by using atomic absorption spectrometry. Supplementing diets with Cr resulted in significantly higher Cr levels in the femurs of rats fed the CEL diet and significantly higher Cr and Fe levels in the rats fed the CEL + PEC diet compared to the rats fed FF diet. Muscle Ca content was significantly lower in the rats fed the CEL + PEC + Cr diet compared to the rats fed FF + Cr diet. The rats consuming the PEC + Cr diet had the highest liver Cr content. The highest kidney Zn content was observed in the rats fed diets containing Cr and one type of fiber. These results indicate that diets containing chromium at elevated dose and fiber have a significant effect on the mineral balance in rat tissues.

## Introduction

Chromium in its trivalent form, Cr(III), is generally regarded as an essential nutrient that regulates carbohydrate, lipid, and protein metabolism by enhancing insulin action [1, 2]. Cr(III) exhibits low toxicity in animals, which may result from its poor transport across cellular membranes [3]. However, there are relatively few articles on the effect of Cr supplementation on the concentration of Cr and other minerals in animal tissues [4–8]. Studies of the distribution of Cr in the tissues of rats indicate that Cr content is greatest in the kidneys. Cr supplementation may also be associated with increased Cr content in the liver, bones, and ovaries. The effects of Cr on tissue mineral concentration show that it has the most significant interaction with Fe [4, 5, 9].

Soluble fiber (pentosans, pectin, gums, and mucilage) performs important physiological functions, e.g., reduces cholesterol level and blood pressure, prevents gastrointestinal problems, protects against the onset of several cancers, and increases mineral bioavailability [10]. The principal potential health benefits of insoluble fibers (cellulose, hemicellulose, and lignin) are related to gastrointestinal transit and constipation. Prospective cohort studies indicate that diets high in insoluble cereal dietary fiber and whole grains might reduce diabetes risk [11]. The relationship between dietary fiber and Ca, Mg, Zn, Mn, Cu, and Fe absorption and balance is controversial, however, with some studies reporting impaired mineral absorption or balance [12, 13] and other studies reporting no change [14] or a positive balance with a high-fiber diet [15–17].

The influence of Cr, when administered in a diet with soluble or insoluble fiber, on the distribution of minerals in the tissues of rats has not been assessed. The present study was undertaken to determine whether diets with cellulose and/or pectin and elevated Cr amount have an interactive effect on the Cr, Ca, Mg, Zn, and Fe content in the tissues of rats.

## Materials and Methods

### Experimental Design

The study was performed on 80 male Buffalo rats (mean initial bodyweight 120 ± 10 g) that were divided into eight groups (ten rats per group, five rats per cage). The rats were housed in standard polypropylene cages with stainless steel wire lids. For 6 weeks, they were fed the following experimental diets: group 1—a fiber-free diet (FF); group 2—a fiber-free diet with chromium (FF + Cr); group 3—a diet with 5 % cellulose (CEL); group 4—a diet with 5 % cellulose and chromium (CEL + Cr); group 5—a diet with 5 % pectin (PEC); group 6—a diet with 5 % pectin and chromium (PEC + Cr); group 7—a diet with 2.5 % cellulose and 2.5 % pectin (CEL + PEC); and group 8—a diet with 2.5 % cellulose, 2.5 % pectin, and chromium (CEL + PEC + Cr) (Table 1). AIN-93 M diets enriched with fat were applied in the study [18]*.* The following diet modifications were made: fat content was increased from 40 to 100 g/kg diet, 90 % of fat was replaced by lard, and cholesterol was added at the level of 2 g/kg diet. The design of the experiment included research on other purposes, i.e., the influence of type of diet on lipid and carbohydrate metabolism [19]. However, the impact of the increased fat content on mineral levels in rat tissue was not studied. Cr was added to the diet with a 0.37 g CrCl_3_ · 6H_2_O/kg mineral mix, yielding a dietary Cr content of 2.53 mg Cr(III)/kg diet. The analytically determined Cr content in low-Cr diets averaged 0.164 mg/kg diet. The apple pectin WEJ-3 F (Pektowin, Jaslo, Poland) and α-cellulose (Sigma-Aldrich, St. Louis, MO, USA; cat. no. C6429) were used. The animals were allowed free access to their food and to distilled water. The rats were kept in cages under a 12-h light cycle at a temperature of 22 ± 1 °C and a humidity level of 55–60 %. The study was approved by the First Local Ethics Commission in Wroclaw (approval no. 20/2006). The intake of Cr, based on an analytical assessment of the diets, is shown in Table 2. Rat urine was collected according to Juturu et al. [20], and the urinary Cr excretion rate was estimated by comparing the amount of Cr excreted in the urine to the Cr intake (Table 2). At the end of the experiment, the rats were food-deprived for 24 h and then anesthetized with Bioketan. Liver, kidney, and thigh muscle samples were collected after the dislocation of the cervical vertebrae, and the femurs were separated and cleaned. All tissues were washed in a cold solution of 0.9 % NaCl, weighed, and then stored at −20 °C until analysis.

**Table 1:** The composition of the experimental diets

Components (g/kg diet)	Experimental diets^a^							
	FF	FF + Cr	CEL	CEL + Cr	PEC	PEC + Cr	CEL + PEC	CEL + PEC + Cr
Wheat starch	615.7	615.7	565.7	565.7	565.7	565.7	565.7	565.7
Casein	145.0	145.0	145.0	145.0	145.0	145.0	145.0	145.0
Sucrose	88.0	88.0	88.0	88.0	88.0	88.0	88.0	88.0
Soybean oil	10.0	10.0	10.0	10.0	10.0	10.0	10.0	10.0
Lard	90.0	90.0	90.0	90.0	90.0	90.0	90.0	90.0
Mineral mixture	35.0	35.0^b^	35.0	35.0^b^	35.0	35.0^b^	35.0	35.0^b^
Vitamin mixture	10.0	10.0	10.0	10.0	10.0	10.0	10.0	10.0
l-cysteine	1.8	1.8	1.8	1.8	1.8	1.8	1.8	1.8
Choline chloride	2.5	2.5	2.5	2.5	2.5	2.5	2.5	2.5
Cholesterol	2.0	2.0	2.0	2.0	2.0	2.0	2.0	2.0
Cellulose	–	–	50.0	50.0	–	–	25.0	25.0
Pectin	–	–	–	–	50.0	50.0	25.0	25.0
Minerals determined in diets (dry mass basis)								
Ca (mg/g)	3.9	3.8	3.8	3.8	3.6	3.2	3.4	3.8
Mg (mg/g)	0.56	0.58	0.57	0.58	0.56	0.57	0.58	0.57
Zn (μg/g)	40	42	39	40	40	41	40	40
Fe (μg/g)	37	36	38	39	40	40	38	39
Cr (μg/g)	0.13	2.33	0.13	2.21	0.21	2.59	0.13	2.30

*FF* a fiber-free diet, *CEL* a diet with 5 % cellulose, *PEC* a diet with 5 % pectin, *CEL + PEC* a diet with 2.5 % cellulose and 2.5 % pectin

^a^The diets differ in fiber content and Cr content

^b^The mineral mix added to the diets with Cr contained 0.37 g CrCl_3_ · 6H_2_O/kg mix (2.53 mg Cr/kg diet, mean value, dry mass basis)

**Table 2:** The Cr intake and minimum absorption of rats in the groups fed experimental diets

	Experimental diets^a^							
	FF	FF + Cr	CEL	CEL + Cr	PEC	PEC + Cr	CEL + PEC	CEL + PEC + Cr
Cr intake (μg/day)	1.69 ± 0.19^A^	36.8 ± 3.16^B^	1.99 ± 0.21^A^	34.5 ± 3.85^B^	3.80 ± 0.63^A^	38.3 ± 1.61^B^	1.96 ± 0.24^A^	34.5 ± 1.63^B^
Cr excretion in urine (μg/day) × 10^−2^	2.02 ± 0.23^A^	2.60 ± 0.31^AB^	3.59 ± 0.21^AB^	6.83 ± 0.44^CDE^	3.84 ± 0.39^ABD^	7.28 ± 0.35^CE^	5.35 ± 0.42^BE^	8.60 ± 2.89^C^
Urinary excretion rate (%)^b^	1.19 ± 0.14^AB^	0.07 ± 0.02^B^	1.84 ± 0.50^AC^	0.19 ± 0.08^BD^	1.01 ± 0.74^ABD^	0.19 ± 0.03^BD^	2.73 ± 1.35^C^	0.25 ± 0.06^BD^

The data are presented as the mean ± SD. The values in the same row that do not share the same superscript letter are significantly different (analysis of variance, *P* < 0.05)

*FF* a fiber-free diet, *CEL* a diet with 5 % cellulose, *PEC* a diet with 5 % pectin, *CEL + PEC* a diet with 2.5 % cellulose and 2.5 % pectin

^a^The diets differ in fiber content and Cr content

^b^Urinary excretion rate (%) = (Cr excreted in rat urine (μg/day) / Cr intake (μg/day)) × 100 %

### Determination of Elements in Rat Tissues and Urine

The samples of tissues and urine (1.0 g) were mineralized with HNO_3_ (65 % Suprapur; Merck, Darmstadt, Germany) in the microwave digestion system Plazmatronika (Wroclaw, Poland) according to the producer’s recommendations. Digested solutions were diluted with deionized water to 10 ml after removing nitrogen dioxide fumes and cooling. Ca, Mg, Fe, and Zn content was determined by flame atomic absorption spectrometry (F-AAS) [21], and Cr content was determined by graphite furnace atomic absorption spectrometry (GF-AAS) [22]. The Perkin Elmer Model 3110 (Norwalk, CT, USA) atomic absorption spectrometer was used for measurements. The accuracy and precision were assessed by determining the levels of Ca, Mg, Fe, Zn, and Cr in the certified reference material (NCS ZC730016 Chicken), which was digested analogously to the samples. The mean recoveries of the certified levels were as follows: Cr—87 %, Ca—89 %, Mg—91 %, Fe—99 %, and Zn—101 %. The femur P content was also determined spectrophotometrically using Scheel’s method [23].

### Statistical Analysis

Data were analyzed using Statistica v. 10.0. An analysis of variance (ANOVA) was performed, followed by Tukey’s post hoc test for intergroup comparison of parametric data. When dealing with nonparametric data, the Kruskal-Wallis test was used. A *P* value less than 0.05 was considered statistically significant.

## Results

The dietary intake and the body mass of the rats did not differ between the groups. The average dietary intake was within the range of 14.6 ± 1.1 to 15.8 ± 1.3 g/day, and the body mass gains were within the range of 100 ± 16 to 119 ± 28 g. The daily Cr excretion in urine was significantly higher in rats fed Cr-supplemented diets with fiber than in other groups. However, the urinary Cr excretion rate was lower in rats that received a Cr-supplemented diet than in the rats that were fed low-Cr diets (Table 2). In rat groups fed fiber-free diets, the amount of excreted Cr per day did not differ significantly despite the different Cr intake. Adding cellulose and/or pectin resulted in significantly higher Cr excretion in rat groups fed Cr-supplemented diets compared to rats fed low-Cr diets.

The mean mineral content in the femurs and thigh muscles of the rats is presented in Table 3. The highest Cr content was observed in the femurs of the rats that ate the CEL + Cr and the CEL + PEC + Cr diets; the lowest Cr levels were found in the rats fed the fiber-free diet, even if the diet was fortified with Cr. The rats that received the PEC + Cr and the CEL + PEC + Cr diets had significantly higher Fe content in the femur than the FF and FF + Cr groups. The femur Zn content was significantly higher in the rats fed a FF diet than in the groups fed diets with fiber. The CEL + PEC diet led to a significantly lower level of P in the femurs of the rats, compared to the FF group. Adding Cr to the FF diet and to the diets with fiber did not yield significant differences in the content of Zn, Mg, or P in the femur. There were no significant differences in the Cr, Fe, and Zn concentrations in the muscles between the groups. The rats fed the CEL + PEC + Cr diets had a significantly lower concentration of muscle Ca compared to the group fed the FF + Cr diet. The CEL + PEC + Cr group was also distinguished from the other groups by having the highest muscle Mg concentration, which was significantly different from the FF group.

**Table 3:** The effects of experimental diets on the mineral content in the femurs and thigh muscles of rats

Mineral	Experimental diets^a^							
	FF	FF + Cr	CEL	CEL + Cr	PEC	PEC + Cr	CEL + PEC	CEL + PEC + Cr
Femur								
Cr (μg/g WM) × 10^−2^	10.1 ± 5.3^A^	13.0 ± 6.9^AB^	18.4 ± 8.1^AB^	28.3 ± 10.4^B^	15.6 ± 6.8^AB^	17.2 ± 9.0^AB^	20.2 ± 7.8^AB^	24.9 ± 9.9^B^
Ca (mg/g WM)	158 ± 21	143 ± 23	149 ± 17	148 ± 20	141 ± 14	135 ± 19	161 ± 20	141 ± 22
Fe (μg/g WM)	59.6 ± 8.2^AC^	59.9 ± 7.3^A^	62.9 ± 6.8^AB^	69.5 ± 5.0^AB^	65.9 ± 7.6^AB^	73.4 ± 8.7^BC^	69.3 ± 6.3^AB^	75.9 ± 13.1^B^
Zn (μg/g WM)	135 ± 8^A^	133 ± 7^AC^	124 ± 6^BC^	123 ± 6^AB^	123 ± 7^B^	124 ± 5^BC^	121 ± 7^B^	127 ± 6^AB^
Mg (mg/g WM)	4.94 ± 0.86	4.54 ± 0.79	5.19 ± 0.99	4.19 ± 0.86	3.68 ± 1.02	3.74 ± 0.83	4.35 ± 0.93	3.68 ± 0.75
P (mg/g WM)	82.3 ± 8.2^AC^	83.4 ± 7.2^A^	76.6 ± 9.2^AB^	79.2 ± 6.6^AB^	68.1 ± 9.6^BC^	72.4 ± 4.0^AB^	65.9 ± 8.7^B^	76.2 ± 9.9^AB^
Muscle								
Cr (μg/g WM) × 10^−2^	16.3 ± 8.9	25.0 ± 9.6	20.8 ± 11.7	32.4 ± 12.8	26.1 ± 9.0	26.7 ± 11.0	20.8 ± 9.8	19.1 ± 7.9
Ca (μg/g WM)	85.1 ± 24.2^AB^	126.0 ± 43.8^A^	72.8 ± 7.6^AB^	82.2 ± 21.8^AB^	93.2 ± 34.3^AB^	72.0 ± 21.9^AB^	70.7 ± 13.6^AB^	60.4 ± 12.9^B^
Fe (μg/g WM)	13.3 ± 2.5	16.5 ± 4.7	12.9 ± 1.6	14.0 ± 3.4	12.7 ± 2.8	12.6 ± 2.0	13.0 ± 2.1	11.7 ± 1.2
Zn (μg/g WM)	11.8 ± 2.1	12.5 ± 3.4	9.4 ± 0.9	11.0 ± 1.9	10.2 ± 2.2	10.2 ± 1.4	10.7 ± 1.4	9.9 ± 1.7
Mg (μg/g WM)	268 ± 23^A^	291 ± 18^AB^	286 ± 10^AB^	283 ± 19^AB^	290 ± 16^AB^	283 ± 13^AB^	278 ± 25^AB^	301 ± 18^B^

The data are presented as the mean ± SD. The values in the same row that do not share the same superscript letter are significantly different (analysis of variance, *P* < 0.05)

*FF* a fiber-free diet, *CEL* a diet with 5 % cellulose, *PEC* a diet with 5 % pectin, *CEL + PEC* a diet with 2.5 % cellulose and 2.5 % pectin, *WM* wet mass

^a^The diets differ in fiber content and Cr content

Table 4 shows the mean mineral content in the livers and kidneys of the rats. In the group that received the PEC + Cr diet, a sixfold to tenfold higher Cr content was observed in the liver compared to the other groups. The lowest Ca, Zn, and Mg content was observed in the livers of the rats fed the PEC diet. The Fe content of the liver did not change significantly following supplementation with cellulose, pectin, and/or Cr. The concentration of Ca in the kidneys was the highest in the group that ate the CEL diet. The addition of pectin or cellulose to diets, especially with Cr, caused significantly higher Zn accumulation in this tissue. Higher Mg content was observed mainly in the groups that received pectin in their diets. The kidney Cr and Fe concentrations were not altered by any of the diets.

**Table 4:** The effects of experimental diets on the Cr, Ca, Fe, Zn, and Mg content in the livers and kidneys of rats

Mineral	Experimental diets^a^							
	FF	FF + Cr	CEL	CEL + Cr	PEC	PEC + Cr	CEL + PEC	CEL + PEC + Cr
Liver								
Cr (μg/g WM) × 10^−2^	3.31 ± 2.51^A^	5.51 ± 2.19^AB^	3.27 ± 1.85^A^	4.51 ± 2.93^A^	3.95 ± 2.46^A^	30.5 ± 10.8^B^	4.20 ± 1.94^AB^	3.03 ± 1.66^AB^
Ca (μg/g WM)	44.1 ± 13.0^A^	54.4 ± 11.3^AB^	56.8 ± 18.3^AB^	47.8 ± 9.8^A^	42.7 ± 8.2^A^	57.7 ± 16.8^AB^	56.1 ± 16.8^AB^	69.0 ± 12.4^B^
Fe (μg/g WM)	104.0 ± 15.3	85.9 ± 15.6	87.5 ± 9.2	90.3 ± 8.0	90.6 ± 10.8	101.0 ± 22.5	93.5 ± 11.3	102.1 ± 8.2
Zn (μg/g WM)	37.0 ± 3.2^A^	33.1 ± 2.9^AB^	35.0 ± 3.3^AB^	35.7 ± 1.1^A^	29.6 ± 3.0^B^	34.5 ± 4.6^AB^	32.6 ± 2.9^AB^	33.5 ± 1.9^AB^
Mg (μg/g WM)	237 ± 28^A^	217 ± 12^AB^	215 ± 21^AB^	209 ± 17^AB^	184 ± 17^B^	231 ± 30^A^	211 ± 30^AB^	221 ± 15^A^
Kidney								
Cr (μg/g WM) × 10^−2^	2.73 ± 1.73	2.67 ± 1.61	3.43 ± 1.85	2.19 ± 0.66	2.44 ± 1.24	2.78 ± 0.71	2.83 ± 1.25	3.65 ± 2.54
Ca (μg/g WM)	49.5 ± 13.5^AB^	37.8 ± 7.2^A^	61.3 ± 9.8^B^	55.6 ± 18.2^AB^	53.5 ± 16.2^AB^	54.5 ± 10.0^AB^	38.4 ± 5.8^A^	43.4 ± 11.3^AB^
Fe (μg/g WM)	46.2 ± 7.2	40.7 ± 9.3	42.2 ± 5.2	44.5 ± 10.1	44.5 ± 5.0	50.0 ± 9.8	41.1 ± 7.6	41.5 ± 7.4
Zn (μg/g WM)	19.5 ± 1.9^AC^	19.7 ± 2.3^AC^	22.5 ± 1.6^BD^	23.7 ± 2.6^B^	22.5 ± 1.3^BD^	24.7 ± 1.6^B^	20.2 ± 1.2^CD^	20.4 ± 1.8^CD^
Mg (μg/g WM)	165 ± 16^AC^	158 ± 9^A^	191 ± 17^BC^	185 ± 20^AB^	196 ± 12^B^	195 ± 19^BC^	172 ± 13^AB^	192 ± 29^BC^

The data are presented as the mean ± SD. The values in the same row that do not share the same superscript letter are significantly different (analysis of variance, *P* < 0.05)

*FF* a fiber-free diet, *CEL* a diet with 5 % cellulose, *PEC* a diet with 5 % pectin, *CEL + PEC* a diet with 2.5 % cellulose and 2.5 % pectin, *WM* wet mass

^a^The diets differ in fiber content and Cr content

## Discussion

The interactive effect of fiber and Cr on mineral distribution in the body has not yet been established. Therefore, studies on the effects of CrCl_3_ intake for a short time (6 weeks), at the level of 2.53 mg elemental Cr/kg diet (250 % of the amount applied to the standard diet for laboratory rodents), were undertaken in rats fed diets with and without pectin and cellulose. The effects were also assessed in rats fed diets with or without fiber containing a low level of Cr (0.164 mg/kg diet), originating naturally from the components of the diet. This low level of Cr in the diet was considered sufficient for rats, as it has been shown that Cr at a tenfold lower level (0.016 mg/kg diet) did not negatively influence the rat organism, at least it did not affect the body composition or glucose metabolism of male Zucker rats [24]. In this study, the urinary Cr excretion rate in Cr-supplemented rats ranged from 0.07 to 0.25 %; in rats fed low-Cr diets, urinary excretion rate ranged from 1.01 to 2.73 %, and the highest value was obtained in rat group fed CEL + PEC diet (Table 2). The influence of dietary fiber on Cr loss has not yet been evaluated. However, the results obtained in this work may suggest the promotive effect of fiber on urinary Cr excretion.

The mean Cr content in the tissues of the experimental rats was highest in the thigh muscles, followed by the femur, the liver, and the kidneys. Cr supplementation (in the form of CrCl_3_) of the fiber-free diet containing 62 % wheat starch had no effect on Cr distribution in the tissues of the rats in comparison with the group fed a FF diet without Cr. In contrast to these findings, Anderson et al. [4] showed that in rats fed diets containing 63 % cornstarch supplemented with different compounds of Cr(III) that provided 5 mg elemental Cr/kg diet, the concentration of Cr in the kidneys was more than ten times higher than in other tissues. In the rats that received a diet with CrCl_3_, these authors found that the Cr content was highest in the kidneys, followed by the lungs, the gastrocnemius muscle, the liver, the spleen, and the heart.

In the present study, Cr supplementation of a diet including cellulose and/or pectin led to a higher Cr and Fe content in the femurs of rats. The increased concentration of Fe in bone as a result of Cr and fiber supplementation may have a potentially beneficial effect. Research conducted by Katsumata et al. [25] demonstrated the role of Fe in maintaining bone mineral density and the mechanical strength of the femur in rats. The role of Cr in the metabolism of bone tissue is poorly understood. It may be related to the anabolic action of insulin. CrPic_3_ has been found to reduce the urinary excretion of hydroxyproline and Ca in postmenopausal women, presumably indicating a reduced rate of bone resorption [26].

In this study, it was observed that adding cellulose and pectin to the diets with Cr promoted lower concentrations of Ca and Fe in skeletal muscle. Chronically low levels of Ca may have a negative impact on muscle contractility [27], while lower Fe reserves may disturb oxygen homeostasis and potentiate erythropoiesis [28].

It was also shown in this study that the Cr content and the Ca content in the liver were very sensitive to supplemental Cr when given together with pectin or pectin and cellulose, respectively. Other studies have demonstrated an increase in the Cr concentration in the livers of rats [4] and pigs [5] fed a diet supplemented with CrPic_3_. The influence of supplemental pectin and Cr given in the form of CrCl_3_ on accumulation of minerals in rat tissues has not yet been examined, so the obtained results could not be compared with published data. The extremely high level of Cr in liver resulting from feeding rats PEC + Cr diet was rather unexpected. Pectin was shown to either prevent or has no influence on metal accumulation in rat tissues. The binding of metal ions to pectin has been demonstrated, which depends on type of pectin as well as metal ion. The effect of pectin was studied mainly for bivalent metals, not for trivalent ones [29, 30]. Acetic acid (the main product of pectin fermentation in lumen) and Cr have been shown to lower blood insulin level [31–33]. As presented in the previous work, a lowering effect on the plasma insulin level of rats was only observed in the Cr-supplemented group that was fed a diet without fiber—however, adding pectin to the Cr-supplemented diet significantly increased the concentration of insulin [19]. These contradictory effects may be the results of metabolic disturbances related to a high-fat diet which could hide the beneficial effects of chromium and pectin. In a study conducted by Król et al. [34], the administration of a high-fat diet with a standard level of fiber (5 % cellulose) also led to increased insulin level in serum of rats whether supplemented with Cr propionate or not (supplementation levels 10 or 50 mg Cr/kg diet). Increased insulin concentration in blood has been shown to decrease the circulating Cr level, as the metal is taken up by insulin-dependent cells via LMWCr formation [35]. The liver is an insulin-sensitive organ, and therefore, supplemental Cr could accumulate in hepatic cells as a consequence of metabolic disturbances.

The results of the present study indicate that the balance of minerals in the kidneys may depend on the type of fiber consumed. According to many studies on rats and other healthy laboratory animals [4, 7, 36], as well as diabetic animals [37], kidney tissue is the most sensitive tissue (or one of the most highly sensitive tissues) to supplemental Cr, although this effect was not shown in the present study. It is not possible to determine the reason for this inconsistency, given the differences in the time of administration, the dosages, the chemical forms of Cr, and the metabolic conditions of the rats.

The observed alterations in tissue mineral distribution might be the results of several mechanisms. Minerals may be bound by dietary fiber, thus decreasing their bioavailability; this mainly concerns insoluble dietary fiber (cellulose) and, as noted in several studies, phytate content mainly potentiated this effect [38]. Except for Zn concentration in the femur, a generally decreased mineral accumulation in rat groups fed diets with purified cellulose was not observed. Rats fed diets supplemented with pectin may absorb more minerals due to the positive influence of carbohydrate fermentation by gut microflora and increased cecum weight [39]. This effect was observed only on Zn and Mg accumulation in the kidneys and, in rats supplemented with Cr and pectin, on Cr level in the liver. This ambiguous influence of soluble fiber on mineral accumulation might be the result of the use of a standard dose of pectin in this study (5 %). In the study by Krejpcio et al. [40], no less than 10 % of soluble fiber (oligofructans) resulted in Mg accumulation in rat bones.

Changes in dietary Cr content could also affect mineral distribution, as shown in several earlier studies [7, 8, 41]. Cr is noted as affecting Fe metabolism mainly through the common transport protein transferrin [42], although no decreased accumulation of Fe was observed in rats supplemented with Cr. Adding PEC to diets supplemented with Cr even improved femur accumulation in rats. Clodfelder et al. [43] did not observe a decrease in Fe levels in kidney and liver tissue from different models of rats either, although there was a trend towards higher Fe kidney accumulation in Sprague Dowley and ZDF rats after Cr supplementation. On the other hand, in a study conducted by Sun et al. [44], the different effects of Cr on Fe metabolism were the result of the chemical form of Cr. Administration of [Cr_3_O(O_2_CCH_2_CH_3_)_6_(H_2_0)_3_]^+^ did not affect Fe concentration in the liver tissue of rats; LMWCr in the diet led to a decrease in Fe content. The interaction between Cr and Fe and rat metabolism may depend on several factors, e.g., the metabolic conditions of rats and the chemical form and the doses of Cr.

In this study, the addition of different types of dietary fiber that show water-holding capacity might inhibit water absorption in the intestine and apparently increase mineral content in organs, although an unequivocal accumulative effect of dietary fiber on tissue mineral concentration was not observed.

## Conclusions

In conclusion, the results of this study indicate that Cr(III) at elevated dose and fiber (i.e., cellulose and pectin) in the diet influenced mineral contents in rat tissues. Effects on mineral distribution differed in particular tissues and depended on the type of dietary fiber in the diet. A further study is warranted to investigate the effects of Cr and fiber ingestion on mineral balance in organism.
